# Institutional Notes and News

**Published:** 1910-01-15

**Authors:** 


### Institutional Notes and News.

**Manchester Royal Eye Extension.**

The Manchester Royal Eye Hospital, next door to the Royal Infirmary, has practically done with the builders by this time. The practical result of this is that university students can congratulate themselves on having separate wards and a separate theatre of their own for ophthalmic cases, and a separate entrance in the side of the infirmary. These additions enable the hospital to serve 160 instead of 110 patients, and those suffering from purulent conjunctivitis have been given a floor to themselves. Special attention, we are glad to learn, has been given to the laundry, and Manchester now possesses two hospitals side by side on which no money has been spared to make efficient.

**A Presentation.**

Last week at Eversfield Hospital, St. Leonards, Mr. Harvey du Cros unveiled a presentation portrait of Dr. Gambier, the founder of the hospital. The portrait, the size of which is 50 inches by 40, is most lifelike, and bears a tablet inscribed as follows: "Portrait of Thomas Gambier, M.D., founder of the Eversfield Chest Hospital, painted by L. Carr Cox, presented by old friends and patients as a token of their affectionate regard. December 1909." Dr. Gambier, in returning thanks, said he did not see that he had done any very great thing. When his friend and colleague, Dr. David Walther, asked him to sit for his portrait, he hesitated because he did not court popularity or publicity. He had lived to work for the benefit of others. When they wanted to do anything for the benefit of others they must ignore self. But it was pressed upon him that it would be to the benefit of the hospital if he yielded, and he did yield, and he found everything had been arranged. They wished to make the hospital still more up to date, and to extend it. They had nearly concluded the purchase of the land opposite, and upon that they hoped to build a nurses' home, an adult patients' department, and a laboratory. They hoped to extend their verandahs, so that they might have more patients sleeping in the open air. Consumption was caused to a great extent through close houses. It was a preventable disease. They had fifty-three patients in the hospital, and they had received since 1884 5,561 patients. The hospital was doing as much good as a Publicity Society of Hastings, because it made the place known as a health resort. The hospital spent £2,000 a year in Hastings, and therefore had reason to expect support.

**The Queen's Sympathy for a Children's Hospital.**

At a meeting on January 6 at the Town Hall, Shoreditch, in the interests of the Queen's Hospital for Children, Hackney Road, the Rev. J. E. Watts-Ditchfield said that only the previous week her Majesty had expressed her deep distress at the financial position of the hospital. This was more particularly gratifying because the attendance at the meeting was very small, which seems to show that her Majesty has more local patriotism and sympathy for the Hackney children than the Hackney people themselves. The Rev. J. E. Watts-Ditchfield said a very large deficit had arisen owing to the enlargement of the hospital. Money usually subscribed found its way into the building fund. A sum of between £5,000 and £6,000 had to be provided or sixty-two beds would have to be closed. Work had greatly increased, the in-patients having risen from 734 in 1908 to 1,941 in 1909, and the out-patients from 30,000 to 34,000. The cost of each patient was less than at any other hospital of the kind in London. The Hon. Rupert Guinness, M.P., moved and Dr. C. Addison seconded a resolution to issue an appeal for support. The latter said that when the question of children's treatment was discussed at St. Bartholomew's in October last the number of children waiting for throat operations made it impossible to take any more, until December 23. We hope that her Majesty's sympathy and example will awaken the latent energies and intelligences of all those who directly or indirectly have benefited from this hospital's work. It stands in a district which is of enormous size, and its patients are, as children, the one class which has no means of helping itself in time of sickness.

